# Epitope mapping of an anti-alpha thalassemia/mental retardation syndrome X-linked monoclonal antibody AMab-6

**DOI:** 10.1016/j.bbrep.2018.07.003

**Published:** 2018-07-13

**Authors:** Mika K. Kaneko, Shinji Yamada, Shunsuke Itai, Yoshikazu Furusawa, Takuro Nakamura, Miyuki Yanaka, Saori Handa, Kayo Hisamatsu, Yoshimi Nakamura, Masato Fukui, Hiroyuki Harada, Yukinari Kato

**Affiliations:** a Department of Antibody Drug Development, Tohoku University Graduate School of Medicine, 2-1 Seiryo-machi, Aoba-ku, Sendai, Miyagi 980-8575, Japan; b Department of Oral and Maxillofacial Surgery, Graduate School of Medical and Dental Sciences, Tokyo Medical and Dental University, 1-5-45, Yushima, Bunkyo-ku, Tokyo 113-8510, Japan; c New Industry Creation Hatchery Center, Tohoku University, 2-1, Seiryo-machi, Aoba-ku, Sendai, Miyagi 980-8575, Japan; d ZENOAQ RESOURCE CO., LTD., 1-1 Tairanoue, Sasagawa, Asaka-machi, Koriyama, Fukushima 963-0196, Japan

**Keywords:** ATRX, alpha-thalassemia/mental-retardation-syndrome-X-linked, mAb, monoclonal antibody, ELISA, enzyme-linked immunosorbent assay, PBS, phosphate-buffered saline, DAB, 3,3-diaminobenzidine tetrahydrochloride, ATRX, AMab-6, Epitope mapping

## Abstract

The alpha-thalassemia/mental-retardation-syndrome-X-linked (ATRX) gene is located on the q arm of the X chromosome. ATRX gene mutations were first discovered in pancreatic neuroendocrine tumors, and subsequently in other cancer subtypes, including gliomas. Molecular subgrouping of gliomas has been more important than conventional histological classifications. Mutations in the isocitrate dehydrogenase (IDH), telomerase reverse transcriptase (TERT) promoter, and ATRX and the codeletion of chromosomes 1p/19q are used as biomarkers for diagnosing the subtypes of diffuse gliomas. We recently developed a sensitive monoclonal antibody (mAb) AMab-6 against ATRX by immunizing mice with recombinant human ATRX. AMab-6 can help to detect ATRX mutations via Western blotting and immunohistochemical analyses. In this study, we characterized the binding epitope of AMab-6 using enzyme-linked immunosorbent assay (ELISA), Western blotting, and immunohistochemical analysis, and found that Gln2368 of ATRX is critical for AMab-6 binding to ATRX. Our findings could be applied to the production of more functional anti-ATRX mAbs.

## Highlights

• ATRX gene is located on the q arm of the X chromosome.
• AMab-6 detected ATRX in Western blot and immunohistochemical analyses sensitively.
• Gln2368 of ATRX is critical for AMab-6-binding to ATRX.
• A blocking peptide completely neutralized AMab-6 reaction by IHC.

## 1 Introduction

The alpha-thalassemia/mental-retardation-syndrome-X-linked (ATRX) gene is located on the q arm of the X chromosome. ATRX gene mutations were first discovered in pancreatic neuroendocrine tumors [1], and subsequently in other cancer subtypes, including gliomas. Gliomas are the most frequently occurring brain tumors and have a heterogeneous molecular background [2]. Molecular subgrouping of gliomas using mutations in isocitrate dehydrogenase (IDH) 1/2, TERT promoter, and ATRX and codeletion of 1p/19q as biomarkers stratifies patients into distinct groups that are more prognostically assessed compared with conventional histological classifications [1], [3], [4], [5]. These molecular subtypes are clinically important because treatment strategies can be planned in accordance with molecular subtype along with the World Health Organization (WHO) tumor grading system. The 2016 WHO Classification of Tumors of the Central Nervous System (2016 WHO CNS) is both conceptually and practically more advanced than the 2007 WHO CNS [6], [7]. The 2016 WHO CNS uses molecular parameters, in addition to histological assessments to define many tumor entities, presents major restructuring of diffuse gliomas, medulloblastomas, and other embryonal tumors; and incorporates new entities that are defined using both histology results and molecular features.

The loss of ATRX mRNA and protein in gliomas is caused by an ATRX mutation. Loss of the ATRX protein can be diagnosed by immunohistochemistry using anti-ATRX antibodies [8], [9]; however, nearly all studies on ATRX protein have used polyclonal antibodies [10] because highly sensitive monoclonal antibodies (mAbs) against human ATRX protein had not been established. Recently, we established a novel anti-ATRX mAb, AMab-6, which is very useful in enzyme-linked immunosorbent assay (ELISA), Western blot, and immunohistochemical analyses [11]. In this study, we characterized the binding epitope of AMab-6 using ELISA, Western blot analysis, and immunohistochemical analyses.

## 2 Materials and methods

### 2.1 Plasmid preparation

Human ATRX cDNA (Accession No. AB102641) encoding amino acids 2273–2413, 2273–2378, 2273–2343, 2273–2308, 2309–2413, 2344–2413, and 2379–2413 were obtained using polymerase chain reaction (PCR) with cDNA derived from human lung cDNA as a template. The primer sets for ATRX are summarized in Supplementary Table 1. ATRXs were subcloned into the expression vector pMAL-c2 (New England Biolabs Inc., Beverly, MA, USA) with MAP (GDGMVPPGIEDK) [12] and PA (GVAMPGAEDDVV) [13] tags using the In-Fusion PCR Cloning Kit (Takara Bio., Inc., Shiga, Japan). Substitution of ATRX amino acids 2273–2413 with either alanine or glycine was performed using a QuikChange Lightning Site-Directed Mutagenesis Kit (Agilent Technologies Inc., Santa Clara, CA, USA). This construct was verified by direct DNA sequencing.

### 2.2 Enzyme-linked immunosorbent assay

Synthesized ATRX peptides using PEPScreen (Sigma-Aldrich Corp., St. Louis, MO, USA) were immobilized on Nunc Maxisorp 96-well immunoplates (Thermo Fisher Scientific Inc., Waltham, MA, USA) at 10 μg/mL for 30 min at 37 °C. After blocking with SuperBlock T20 (PBS) Blocking Buffer (Thermo Fisher Scientific Inc.), the plates were incubated with 10 μg/mL purified AMab-6 followed by a 1:2000 dilution of peroxidase-conjugated anti-mouse IgG (Agilent Technologies Inc.). The enzymatic reaction was conducted using 1-Step Ultra TMB-ELISA (Thermo Fisher Scientific Inc.). Optical density was measured at 655 nm using an iMark Microplate Reader (Bio-Rad Laboratories, Inc., Berkeley, CA, USA). These reactions were performed at 37 °C using a total sample volume of 50–100 μL.

### 2.3 Western blot analyses

Competent *Escherichia coli* TOP-10 cells (Thermo Fisher Scientific Inc.) were transformed and cultured overnight at 37 °C in LB medium (Thermo Fisher Scientific Inc.) containing 100 μg/mL ampicillin (FUJIFILM Wako Pure Chemical Corporation, Osaka, Japan). Cell pellets were resuspended in phosphate buffered solution with 1% Triton X-100 and 50 μg/mL aprotinin (Sigma-Aldrich Corp.). Lysates (10 μg) were boiled in sodium dodecyl sulfate sample buffer (Nacalai Tesque, Inc., Kyoto, Japan). The proteins were electrophoresed on 5–20% polyacrylamide gels (FUJIFILM Wako Pure Chemical Corporation) and transferred onto a polyvinylidene difluoride (PVDF) membrane (Merck KGaA, Darmstadt, Germany). After blocking with 4% skim milk (Nacalai Tesque, Inc.), the membrane was first incubated with AMab-6 [11] or NZ-1 (anti-PA tag) [13] and then with peroxidase-conjugated anti-mouse or anti-rat antibody (1:1000 diluted; Agilent Technologies Inc.) and developed using the Pierce Western Blotting Substrate Plus (Thermo Fisher Scientific Inc.) or the ImmunoStar LD Chemiluminescence Reagent (FUJIFILM Wako Pure Chemical Corporation) using a Sayaca-Imager (DRC Co. Ltd., Tokyo, Japan).

### 2.4 Immunohistochemical analyses

This study examined one patient with oral cancer who underwent surgery at Tokyo Medical and Dental University. The Tokyo Medical and Dental University Institutional Review Board reviewed and approved the use of the human cancer tissues, and written informed consent was obtained from the patient. Histological sections (4-μm thick) were directly autoclaved for 20 min in citrate buffer (pH 6.0; Nichirei Biosciences, Inc., Tokyo, Japan). After blocking with SuperBlock T20 (PBS) Blocking Buffer (Thermo Fisher Scientific Inc.), the sections were incubated with 5 μg/mL AMab-6 or 5 μg/mL AMab-6 plus 5 μg/mL peptides for 1 h at room temperature and treated using an EnVision+ Kit (Agilent Technologies Inc.) for 30 min. Color was developed using 3,3′-diaminobenzidine tetrahydrochloride (DAB; Agilent Technologies Inc.) for 2 min, and counterstained with hematoxylin (FUJIFILM Wako Pure Chemical Corporation).

## 3 Results and discussion

Immunohistochemistry is a robust and widely available method used to assess genetic changes at the molecular level using defined protocols and materials [14], [15]. The important molecules for subtype diagnosis of diffuse gliomas are mutations of IDH1/2, TERT promoter, and ATRX and the codeletion of 1p/19q among the many molecular parameters [16]. Mutations of IDH1/2 [14] and ATRX [8], [10] can be accurately detected using this method. We recently developed both anti-mutated IDH mAbs [17] and an anti-ATRX mAb, AMab-6 [11]. Several mAbs against IDH mutants include HMab-1/HMab-2 against IDH1-R132H and multi-specific mAbs MsMab-1/MsMab-2 against IDH1/2 mutations [18], [19], [20], [21].

**Fig. 1:**
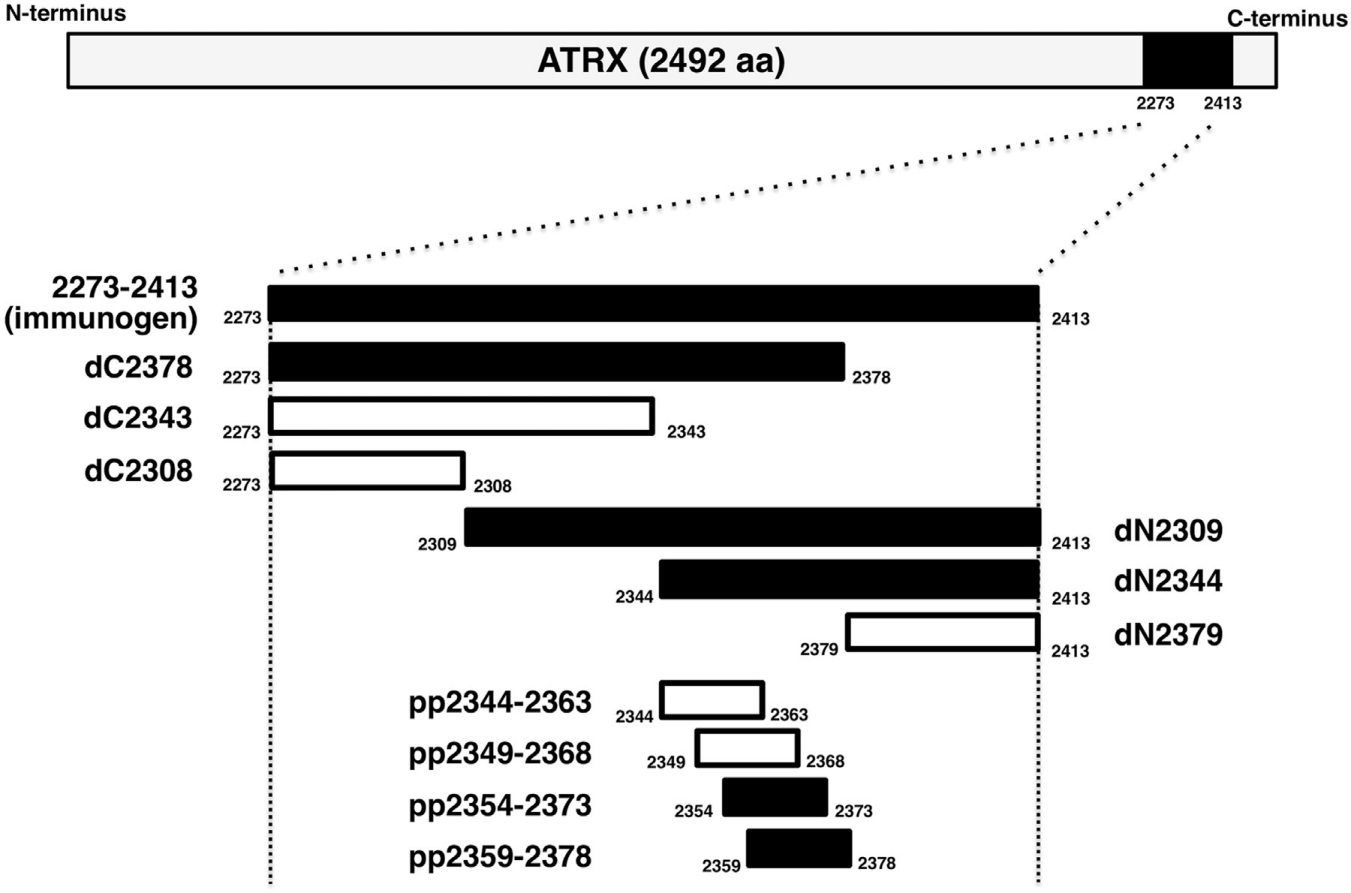
**Production of ATRX deletion mutants.** Three ATRX C-terminal deletion mutants and three ATRX N-terminal deletion mutants were produced. Four ATRX peptides were also synthesized. Black bars, the deletion mutants or synthesized peptides, which were detected by AMab-6; white bar, the deletion mutants or synthesized peptides, which were not detected by AMab-6.

**Fig. 2:**
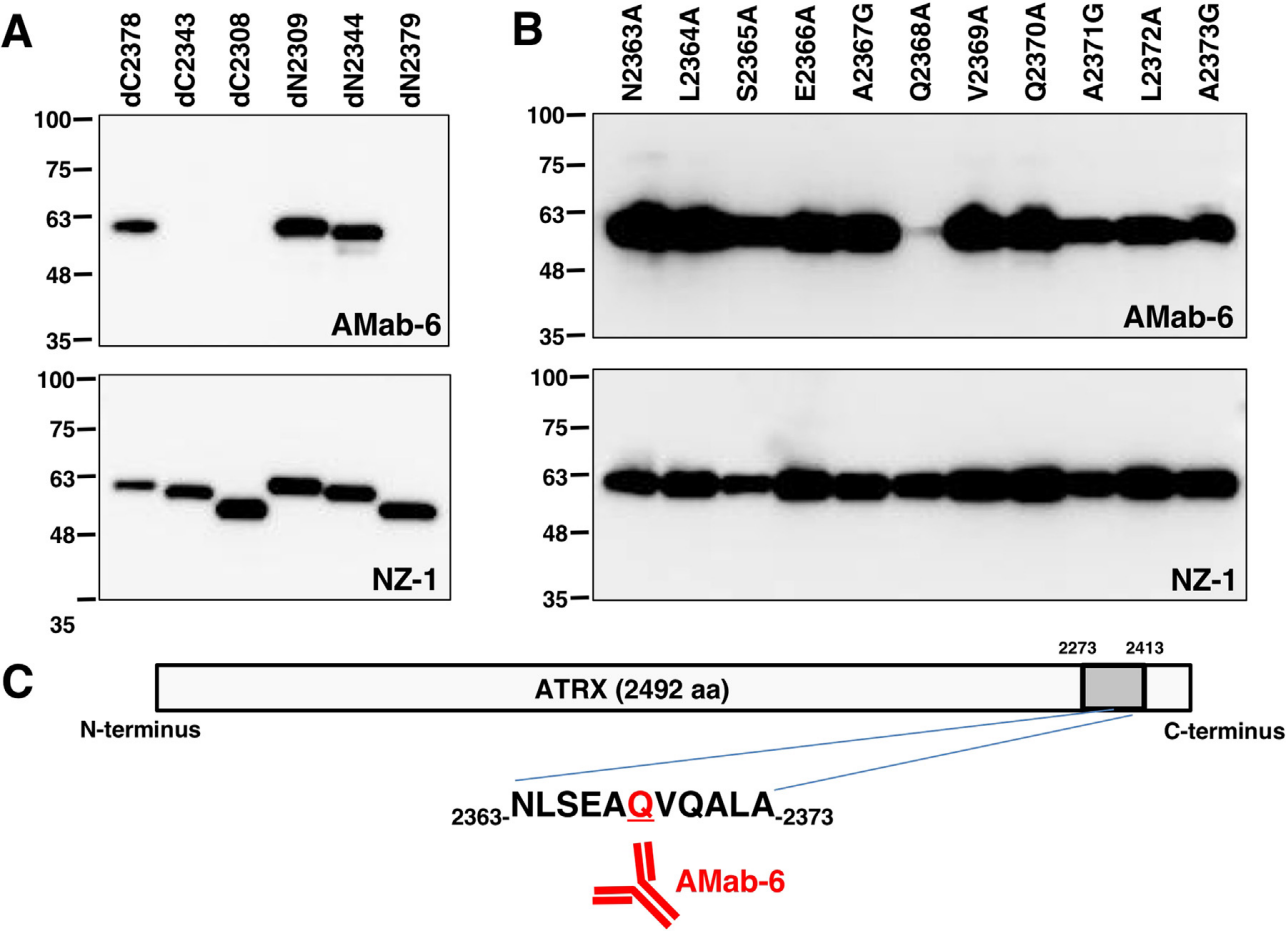
**Epitope mapping of AMab-6 using deletion mutants and point mutants of ATRX.** Cell lysates (10 μg) of deletion mutants (A) and point mutants (B) were electrophoresed and proteins were transferred onto PVDF membranes. After blocking, membranes were incubated with 1 μg/mL of AMab-6 or 1 μg/mL of anti-PA tag (clone: NZ-1) followed by peroxidase-conjugated anti-mouse or rat IgG.

As shown in Fig. 1, we produced three C-terminal deletion mutants (dC2378, dC2343, and dC2308) and three N-terminal deletion mutants (dN2309, dN2344, and dN2379). Western blot analysis demonstrated that AMab-6 detected dC2378, dN2309, and dN2344 but not dC2343, dC2308, and dN2379 (Fig. 2A), indicating that the N-terminus of the AMab-6-epitope exits between amino acids 2344 and 2379, and the C-terminus of the AMab-6-epitope exits between amino acids 2343 and 2378. Next, we produced the following four peptides: pp2344–2363 (ATRX amino acids 2344–2363), pp2349–2368 (ATRX amino acids 2349–2368), pp2354–2373 (ATRX amino acids 2354–2373), and pp2359–2378 (ATRX amino acids 2359–2378) as depicted in Fig. 1. ELISA demonstrated that AMab-6 detected pp2354–2373 and pp2359–2378, and did not react with pp2344–2363 or pp2349–2368 (Table 1).

**Table 1:** Determination of AMab-6 Epitope by ELISA.

Peptide	Sequence	AMab-6
2344–2363	AVRIQPLEDIISAVWKENMN	−
2349–2368	PLEDIISAVWKENMNLSEAQ	−
2354–2373	ISAVWKENMNLSEAQVQALA	＋＋＋
2359–2378	KENMNLSEAQVQALALSRQA	＋＋＋
K2359A	AENMNLSEAQVQALALSRQA	＋＋＋
E2360A	KANMNLSEAQVQALALSRQA	＋＋＋
N2361A	KEAMNLSEAQVQALALSRQA	＋＋＋
M2362A	KENANLSEAQVQALALSRQA	＋＋＋
N2363A	KENMALSEAQVQALALSRQA	＋＋＋
L2364A	KENMNASEAQVQALALSRQA	＋＋＋
S2365A	KENMNLAEAQVQALALSRQA	＋＋＋
E2366A	KENMNLSAAQVQALALSRQA	＋＋＋
A2367G	KENMNLSEGQVQALALSRQA	＋＋＋
Q2368A	KENMNLSEAAVQALALSRQA	＋
V2369A	KENMNLSEAQAQALALSRQA	＋＋＋
Q2370A	KENMNLSEAQVAALALSRQA	＋＋＋
A2371G	KENMNLSEAQVQGLALSRQA	＋＋＋
L2372A	KENMNLSEAQVQAAALSRQA	＋＋＋
A2373G	KENMNLSEAQVQALGLSRQA	＋＋＋

＋＋＋, OD655 ≧ 1.0; ＋＋, 0.6 ≦ OD655＜1.0.

＋, 0.1 ≦ OD655＜0.6; -, OD655＜0.1.

We further synthesized the following 15 peptides of ATRX point mutations: K2359A, E2360A, N2361A, M2362A, N2363A, L2364A, S2365A, E2366A, A2367G, Q2368A, V2369A, Q2370A, A2371G, L2372A, and A2373G (Table 1). ELISA demonstrated that AMab-6 reacted strongly with nearly all point mutants; however, it reacted very weakly with Q2368A, indicating that Gln2368 is important for AMab-6 binding to ATRX protein.

We produced the following 11 ATRX point mutants: N2363A, L2364A, S2365A, E2366A, A2367G, Q2368A, V2369A, Q2370A, A2371G, L2372A, and A2373G. Western blotting demonstrated that AMab-6 did not detect mutant Q2368A (Fig. 2B), confirming that Gln2368 is important for AMab-6 binding to ATRX protein.

**Fig. 3:**
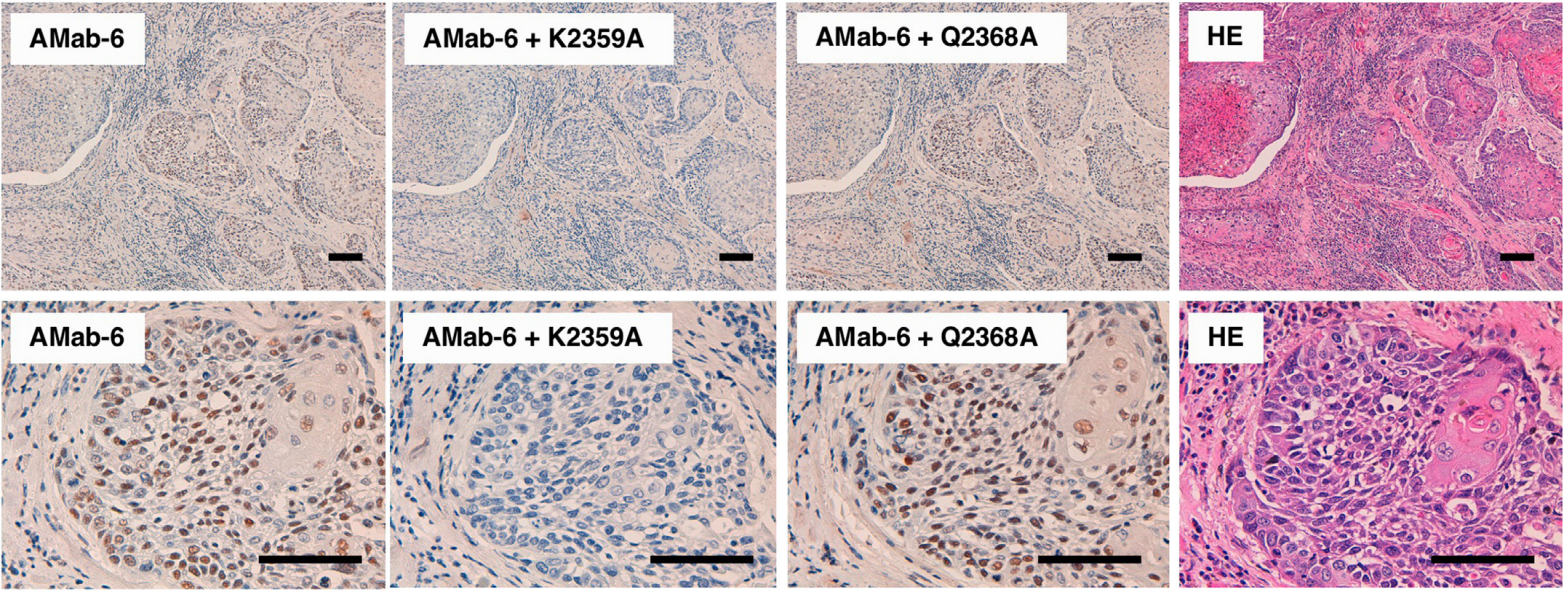
**Immunohistochemistry using oral cancer tissue.** Oral cancer tissues were autoclaved for 20 min in a citrate buffer. Sections were then incubated with 5 μg/mL AMab-6 or 5 μg/mL AMab-6 plus 5 μg/mL peptides and stained using an EnVision+ kit. Color development was performed using 3,3′-diaminobenzidine tetrahydrochloride. Sections were then counterstained with hematoxylin. Notes: scale bar = 100 µm; HE, hematoxylin and eosin.

ATRX mutation has been reported in gliomas in which the ATRX protein is not detected by anti-ATRX antibodies via immunohistochemistry [8], [9], [11], [22]. In contrast, the ATRX protein is usually detected in nearly all cancers, including oral cancers, via immunohistochemistry using anti-ATRX antibodies because ATRX mutations have not been reported in those cancers [22]. In this study, we first performed immunohistochemistry against oral cancers using AMab-6. The nuclei of oral cancer cells were strongly stained by AMab-6 (Fig. 3). We next performed a blocking assay using immunohistochemistry against oral cancers. We found that the reaction of AMab-6 was neutralized by K2359A peptide (Fig. 3). In contrast, the Q2368A peptide did not block the reaction of AMab-6, thereby confirming the results of epitope mapping using ELISA and Western blotting.

In conclusion, Gln2368 of ATRX is critical for AMab-6 binding. Our findings can be applied for the production of more functional anti-ATRX mAbs.

## Funding

This research was supported in part by AMED under Grant numbers: JP18am0101078 (Y.K.), JP18am0301010 (Y.K.), and JP18ae0101028 (Y.K.), and by JSPS KAKENHI Grant Number 17K07299 (M.K.K.) and Grant Number 16K10748 (Y.K.).

## Conflict of interest

The authors declare no conflicts of interest involving this article.
