# Supplementary material for: Epitope mapping of an anti-alpha thalassemia/mental retardation syndrome X-linked monoclonal antibody AMab-6
Source: Biochem Biophys Rep. 2018 Jul 13;15:76–80. doi: 10.1016/j.bbrep.2018.07.003 (PMC6068083; doi:10.1016/j.bbrep.2018.07.003)
Supplement: Supplementary file 2 — Supplementary material [file mmc2.docx]

Supplementary table1

| Primer name | Sequences |
| --- | --- |
| InF.EcoRI-ATRX_F2273 | AAGGATTTCAGAATTCGCAGCTTGGGCTGAGTA |
| InFr. ATRX_R2413-EcoRI | TGCCGTCTCCGAATTCCCTTCTGTTCATAAGTATTC |
| InFr.ATRX_R2378-EcoRI | CGTTAGCATTAAGTAGACAAGAATTCGGAGACGGCA |
| InFr.ATRX_R2343-EcoRI | GAAGCAACAAACAGTGTGGAATTCGGAGACGGCA |
| InFr.ATRX_R2308-EcoRI | TTTCAACTCTCAAACTCCTGAATTCGGAGACGGCA |
| InF.EcoRI-ATRX-F2309 | AAGGATTTCAGAATTCTATATTCCTTTCAATTTGGG |
| InF.EcoRI-ATRX-F2344 | AAGGATTTCAGAATTCACAGCAGTGAGGATTCAA |
| InF.EcoRI-ATRX-F2379 | AAGGATTTCAGAATTCGCCAGCCAGGAGCTTGA |
